# Supplementary material for: Metabolic Effects of Bovine Milk Oligosaccharides on Selected Commensals of the Infant Microbiome—Commensalism and Postbiotic Effects
Source: Metabolites. 2020 Apr 24;10(4):167. doi: 10.3390/metabo10040167 (PMC7240951; doi:10.3390/metabo10040167)
Supplement: Supplementary file 1 [file metabolites-10-00167-s001.zip › Supplementary material/Supplementary material_updated.pdf]

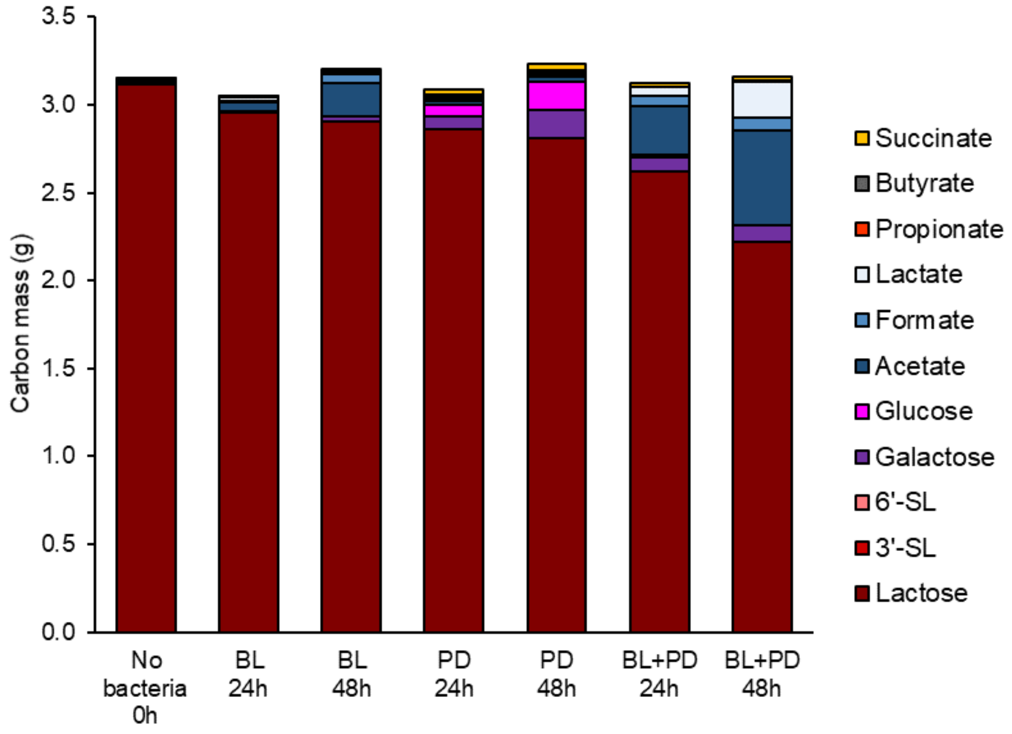


*Supplementary Figure 1 Carbon mass balance after 24h or 48h fermentation of 1 % LAC by Bifidobacterium longum* subsp. *longum (BL), Parabacteroides distasonis (PD) or coculture (BL+PD). Carbon mass was calculated as: g substrate × number of C-atoms × 12 divided by the molecular weight of substrate. Each stacked segment of the bars represent a mean value from three biological replicates. LAC: lactose.*

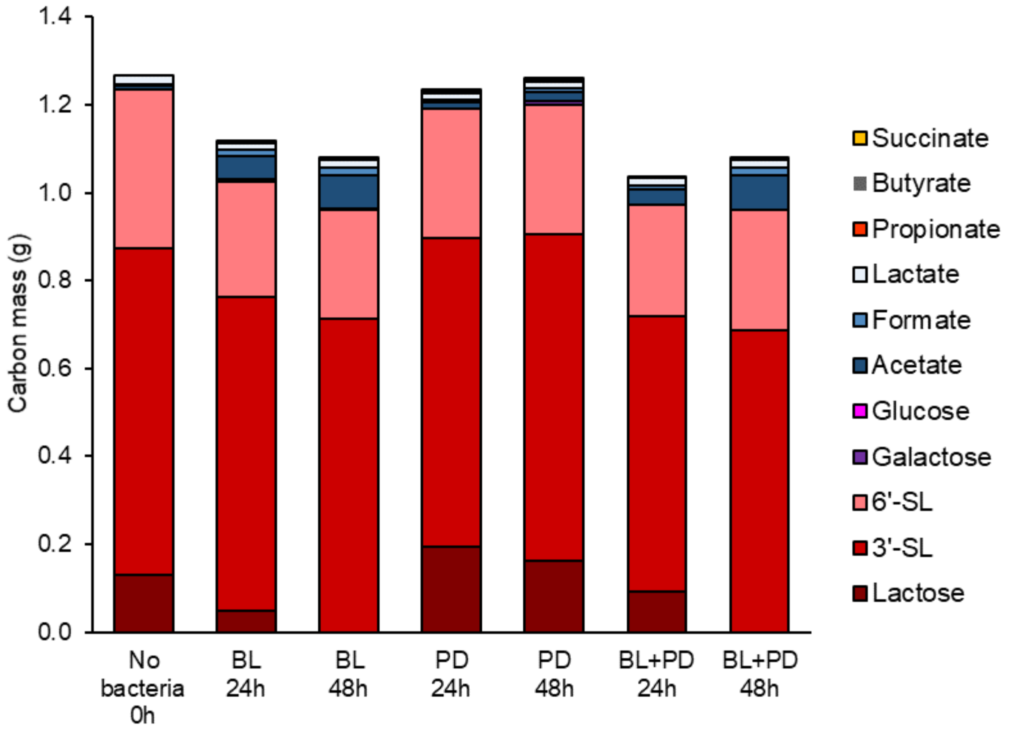


*Supplementary Figure 2 Carbon mass balance after 24h or 48h fermentation of 1 % BMO by Bifidobacterium longum* subsp. *longum (BL), Parabacteroides distasonis (PD) or coculture (BL+PD). Carbon mass was calculated as: g substrate × number of C-atoms × 12 divided by the molecular weight of substrate. Each stacked segment of the bars represent a mean value from three biological replicates. BMO: bovine milk oligosaccharides.*

*Supplementary Table 1 Carbon mass after 24h or 48h fermentation of 1 % LAC by Bifidobacterium longum* subsp. *longum (BL), Parabacteroides distasonis (PD) or coculture (BL+PD). Carbon mass was calculated as: g substrate × number of C-atoms × 12 divided by the molecular weight of substrate. The data represent mean and SD is the standard deviation obtained from three biological replicates. LAC: lactose*

| Bacteria | No bacteria | | BL | | | | PD | | | | BL+PD | | | |
|---|---|---|---|---|---|---|---|---|---|---|---|---|---|---|
| Time | 0h | | 24h | | 48h | | 24h | | 48h | | 24h | | 48h | |
| | mean | SD | mean | SD | mean | SD | mean | SD | mean | SD | mean | SD | mean | SD |
| Succinate | 0.003 | 0.000 | 0.004 | 0.000 | 0.004 | 0.000 | 0.027 | 0.007 | 0.037 | 0.004 | 0.022 | 0.008 | 0.024 | 0.009 |
| Butyrate | 0.001 | 0.001 | 0.000 | 0.000 | 0.000 | 0.000 | 0.002 | 0.003 | 0.002 | 0.003 | 0.002 | 0.003 | 0.000 | 0.000 |
| Propionate | 0.000 | 0.000 | 0.002 | 0.000 | 0.001 | 0.001 | 0.005 | 0.001 | 0.006 | 0.001 | 0.004 | 0.000 | 0.004 | 0.001 |
| Lactate | 0.016 | 0.002 | 0.018 | 0.002 | 0.021 | 0.003 | 0.016 | 0.000 | 0.017 | 0.000 | 0.046 | 0.010 | 0.204 | 0.046 |
| Formate | 0.002 | 0.000 | 0.013 | 0.007 | 0.047 | 0.033 | 0.011 | 0.001 | 0.013 | 0.001 | 0.060 | 0.002 | 0.077 | 0.006 |
| Acetate | 0.006 | 0.001 | 0.048 | 0.023 | 0.189 | 0.126 | 0.024 | 0.002 | 0.029 | 0.001 | 0.281 | 0.019 | 0.538 | 0.050 |
| GLU | 0.006 | 0.008 | 0.000 | 0.000 | 0.001 | 0.002 | 0.064 | 0.011 | 0.159 | 0.056 | 0.009 | 0.008 | 0.000 | 0.000 |
| GAL | 0.000 | 0.000 | 0.008 | 0.003 | 0.027 | 0.020 | 0.078 | 0.027 | 0.160 | 0.054 | 0.087 | 0.008 | 0.091 | 0.012 |
| 6SL | 0.000 | 0.000 | 0.000 | 0.000 | 0.000 | 0.000 | 0.000 | 0.000 | 0.000 | 0.000 | 0.000 | 0.000 | 0.000 | 0.000 |
| 3SL | 0.000 | 0.000 | 0.000 | 0.000 | 0.000 | 0.000 | 0.000 | 0.000 | 0.000 | 0.000 | 0.000 | 0.000 | 0.000 | 0.000 |
| LAC | 3.108 | 0.613 | 2.952 | 0.083 | 2.909 | 0.119 | 2.866 | 0.120 | 2.808 | 0.173 | 2.621 | 0.035 | 2.218 | 0.074 |
| Sum of carbon mass in g | 3.142 | | 3.044 | | 3.200 | | 3.092 | | 3.231 | | 3.132 | | 3.155 | |

*Supplementary Table 2 Carbon mass after 24h or 48h fermentation of 1 % BMO by Bifidobacterium longum* subsp. *longum (BL), Parabacteroides distasonis (PD) or coculture (BL+PD). Carbon mass was calculated as: g substrate × number of C-atoms × 12 divided by the molecular weight of substrate. The data represent mean and SD is the standard deviation obtained from three biological replicates. BMO: Bovine milk oligosaccharides*

| Bacteria | No bacteria | | BL | | | | PD | | | | BL+PD | | | |
|---|---|---|---|---|---|---|---|---|---|---|---|---|---|---|
| Time | 0h | | 24h | | 48h | | 24h | | 48h | | 24h | | 48h | |
| | mean | SD | mean | SD | mean | SD | mean | SD | mean | SD | mean | SD | mean | SD |
| Succinate | 0.004 | 0.001 | 0.004 | 0.000 | 0.004 | 0.000 | 0.004 | 0.000 | 0.006 | 0.002 | 0.004 | 0.000 | 0.004 | 0.000 |
| Butyrate | 0.000 | 0.000 | 0.000 | 0.000 | 0.000 | 0.000 | 0.000 | 0.000 | 0.000 | 0.000 | 0.000 | 0.000 | 0.000 | 0.000 |
| Propionate | 0.000 | 0.000 | 0.000 | 0.000 | 0.000 | 0.000 | 0.002 | 0.003 | 0.003 | 0.006 | 0.000 | 0.000 | 0.002 | 0.003 |
| Lactate | 0.018 | 0.004 | 0.016 | 0.000 | 0.017 | 0.001 | 0.015 | 0.000 | 0.016 | 0.000 | 0.017 | 0.001 | 0.018 | 0.001 |
| Formate | 0.002 | 0.000 | 0.014 | 0.007 | 0.018 | 0.002 | 0.006 | 0.001 | 0.007 | 0.003 | 0.009 | 0.005 | 0.019 | 0.002 |
| Acetate | 0.007 | 0.001 | 0.054 | 0.032 | 0.077 | 0.003 | 0.015 | 0.002 | 0.020 | 0.008 | 0.034 | 0.025 | 0.078 | 0.004 |
| GLU | 0.000 | 0.000 | 0.000 | 0.000 | 0.000 | 0.000 | 0.000 | 0.000 | 0.000 | 0.000 | 0.000 | 0.000 | 0.000 | 0.000 |
| GAL | 0.000 | 0.000 | 0.004 | 0.003 | 0.002 | 0.003 | 0.000 | 0.000 | 0.009 | 0.009 | 0.000 | 0.000 | 0.001 | 0.001 |
| 6SL | 0.322 | 0.046 | 0.262 | 0.023 | 0.250 | 0.025 | 0.295 | 0.047 | 0.295 | 0.023 | 0.256 | 0.017 | 0.271 | 0.032 |
| 3SL | 0.667 | 0.052 | 0.712 | 0.052 | 0.712 | 0.031 | 0.701 | 0.061 | 0.745 | 0.011 | 0.627 | 0.055 | 0.690 | 0.051 |
| LAC | 0.115 | 0.009 | 0.049 | 0.085 | 0.000 | 0.000 | 0.194 | 0.024 | 0.161 | 0.011 | 0.093 | 0.084 | 0.000 | 0.000 |
| Sum of carbon mass in g | 1.135 | | 1.114 | | 1.080 | | 1.233 | | 1.263 | | 1.039 | | 1.083 | |

*Supplementary Table 3 Carbon mass after 24h or 48h fermentation of 1 % BMO+LAC by Bifidobacterium longum* subsp. *longum (BL), Parabacteroides distasonis (PD) or coculture (BL+PD). Carbon mass was calculated as: g substrate × number of C-atoms × 12 divided by the molecular weight of substrate. The data represent mean and SD is the standard deviation obtained from three biological replicates. BMO: Bovine milk oligosaccharides, LAC: lactose*

| Bacteria | No bacteria | | BL | | | | PD | | | | BL+PD | | | |
|---|---|---|---|---|---|---|---|---|---|---|---|---|---|---|
| Time | 0h | | 24h | | 48h | | 24h | | 48h | | 24h | | 48h | |
| | mean | SD | mean | SD | mean | SD | mean | SD | mean | SD | mean | SD | mean | SD |
| Succinate | 0.003 | 0.001 | 0.005 | 0.000 | 0.004 | 0.002 | 0.021 | 0.023 | 0.063 | 0.029 | 0.010 | 0.005 | 0.007 | 0.010 |
| Butyrate | 0.000 | 0.000 | 0.000 | 0.000 | 0.000 | 0.000 | 0.000 | 0.000 | 0.000 | 0.000 | 0.000 | 0.000 | 0.000 | 0.000 |
| Propionate | 0.000 | 0.000 | 0.000 | 0.000 | 0.000 | 0.000 | 0.063 | 0.073 | 0.147 | 0.042 | 0.018 | 0.025 | 0.017 | 0.025 |
| Lactate | 0.015 | 0.003 | 0.090 | 0.008 | 0.091 | 0.041 | 0.015 | 0.001 | 0.019 | 0.001 | 0.117 | 0.049 | 0.482 | 0.094 |
| Formate | 0.002 | 0.000 | 0.052 | 0.002 | 0.060 | 0.023 | 0.023 | 0.015 | 0.039 | 0.003 | 0.062 | 0.018 | 0.067 | 0.024 |
| Acetate | 0.005 | 0.001 | 0.317 | 0.013 | 0.346 | 0.129 | 0.050 | 0.032 | 0.090 | 0.007 | 0.384 | 0.036 | 0.833 | 0.073 |
| GLU | 0.000 | 0.000 | 0.006 | 0.006 | 0.000 | 0.000 | 0.081 | 0.105 | 0.294 | 0.161 | 0.000 | 0.000 | 0.000 | 0.000 |
| GAL | 0.000 | 0.000 | 0.058 | 0.008 | 0.054 | 0.018 | 0.129 | 0.160 | 0.371 | 0.127 | 0.047 | 0.044 | 0.052 | 0.041 |
| 6SL | 0.140 | 0.006 | 0.139 | 0.015 | 0.114 | 0.055 | 0.129 | 0.004 | 0.120 | 0.005 | 0.131 | 0.027 | 0.118 | 0.020 |
| 3SL | 0.321 | 0.059 | 0.359 | 0.025 | 0.268 | 0.104 | 0.334 | 0.010 | 0.326 | 0.033 | 0.334 | 0.008 | 0.348 | 0.000 |
| LAC | 1.550 | 0.376 | 1.064 | 0.012 | 0.854 | 0.307 | 1.223 | 0.423 | 0.586 | 0.480 | 0.979 | 0.112 | 0.196 | 0.093 |
| Sum of carbon mass in g | 2.035 | | 2.090 | | 1.792 | | 2.068 | | 2.055 | | 2.082 | | 2.121 | |

*Supplementary Table 4 Growth (24h) (log copy numbers per mL culture) of Parabacteroides distasonis (PD), Bifidobacterium longum subsp. longum (BL) and Clostridium perfringens (CP) in 1 % treatments of bovine milk oligosaccharides (BMO), lactose (LAC), a combination of the two (BMO+LAC) or minimal media with no carbohydrate (MM). C. perfringens was grown on spent media from B. longum (BL,CP) and P. distasonis (PD,CP). The data represent mean and standard error, calculated from two biological replicates and two technical replicates each. Significant effect of carbon source on logCFU/mL culture was tested by ANOVA and Tukey HSD was used for multiple comparisons between groups. $P \leq 0.05$ indicates significant differences and different letters in each row indicate significant differences*.

| | BMO | | BMO+LAC | | LAC | | MM | | p-value |
|---|---|---|---|---|---|---|---|---|---|
| | *Mean* | *SE* | *Mean* | *SE* | *Mean* | *SE* | *Mean* | *SE* | |
| BL,- | 6.00 | 0.278 | 7.27 | 0.278 | 6.56 | 0.278 | 6.00 | 0.278 | 0.0855 |
| PD,- | 7.06ab | 0.467 | 8.76b | 0.467 | 7.83ab | 0.467 | 6.00a | 0.467 | 0.0170 |
| CP,- | 8.11b | 0.0787 | 7.55a | 0.0787 | 8.05b | 0.0787 | 7.37a | 0.0787 | 0.0063 |
| BL,CP | 8.22 | 0.538 | 6.65 | 0.538 | 6.86 | 0.538 | 7.15 | 0.538 | 0.3076 |
| PD,CP | 8.25b | 0.146 | 7.56ab | 0.146 | 7.76ab | 0.146 | 7.37a | 0.146 | 0.0486 |
